# Examining the Impact of a Codeveloped Multicomponent Mobile eHealth Lifestyle Intervention on Physical Activity and Its Association With Gestational Weight Gain in Underserved Women: A Statewide Randomized Controlled Trial

**DOI:** 10.2196/73962

**Published:** 2025-11-11

**Authors:** Hannah E Cabre, Kaja Falkenhain, Abby D Altazan, Emily W Flanagan, Chelsea L Kracht, Joshua R Sparks, Maryam Kebbe, Emily K Woolf, Daniel S Hsia, L. Anne Gilmore, Robbie Beyl, John W Apolzan, Leanne M Redman

**Affiliations:** 1 Pennington Biomedical Research Center Baton Rouge, LA United States; 2 Department of Internal Medicine The University of Kansas Medical Center Lawrence, KS United States; 3 Naval Health Research Center San Diego, CA United States; 4 University of New Brunswick Fredericton, NB Canada; 5 Department of Pediatrics Emory University Atlanta, GA United States; 6 Department of Clinical Nutrition The University of Texas Southwestern Medical Center Houston, TX United States

**Keywords:** gestation, maternal health, obesity, overweight, pregnancy, movement behaviors

## Abstract

**Background:**

Underserved pregnant women have a greater risk of excessive or inadequate gestational weight gain (GWG) and adverse perinatal outcomes. In the United States, the Special Supplemental Nutrition Program for Women, Infants, and Children (WIC) provides supplemental nutrition and is uniquely positioned to deliver equitable interventions that support recommended GWG. Yet to date, no randomized controlled trials have evaluated behavioral strategies for managing GWG in this setting.

**Objective:**

The primary objective was to examine the effects of a statewide randomized multicomponent mobile eHealth lifestyle intervention trial on change in physical activity and sedentary time across pregnancy. The secondary objective was to explore associations between changes in physical activity, sedentary time, and GWG.

**Methods:**

A total of 351 pregnant women were recruited from the Louisiana WIC clinics and were randomly assigned to a multicomponent mobile eHealth intervention for GWG management (N=179) or usual care (N=172; standard in-person WIC care) prior to 16 weeks of gestation. The multicomponent mobile intervention included daily weighing, step tracking, counseling, exercise videos, health coach interactions, and social support. For the first objective, physical activity, including movement duration and movement context, and sedentary time were assessed at baseline (early pregnancy) and at the end of the intervention (late pregnancy) using accelerometry and the Pregnancy Physical Activity Questionnaire. For the second objective, GWG was determined based on weight collected at study visits in early and late pregnancy. Linear mixed models assessed intervention effects on physical activity and GWG.

**Results:**

Both the Intervention Group and the Usual Care Group significantly increased sedentary time from early to late pregnancy (adjusted effect estimate [95% CI] 62 minutes per day (42-83), *P*<.001 and 52 minutes per day (31-72), *P*<.001, respectively). Both the Intervention Group and the Usual Care Group significantly decreased moderate activity (–13 minutes per day (–20 to –6), *P*<.001 and –10 minutes per day (–17 to –3), *P*=.01, respectively) and total moderate to vigorous physical activity (–14 minutes per day (–21 to –7), *P*<.001 and –10 minutes per day (–18 to –3), *P*=.01, respectively) from early to late pregnancy. For the Pregnancy Physical Activity Questionnaire, the Intervention Group reported an increase in sports participation across pregnancy compared with the Usual Care Group (+4 metabolic equivalent task (MET)–hours per week (2-7); *P*=.002). There were no associations between physical activity (–7 g (–32 to 18), *P*=.57) or sedentary time measures (4 g (–4 to 12), *P*=.31) and GWG.

**Conclusions:**

The first of its kind mobile eHealth multicomponent behavioral lifestyle intervention that included guidance to increase physical activity toward national guidelines did not meaningfully impact physical activity outcomes in pregnant women who were enrolled in Louisiana WIC.

**Trial Registration:**

ClinicalTrials.gov NCT04028843; https://www.clinicaltrials.gov/study/NCT04028843

**International Registered Report Identifier (IRRID):**

RR2-10.2196/18211

## Introduction

Physical activity is an important aspect of a healthy lifestyle, particularly during pregnancy. Regular physical activity during pregnancy is associated with lower gestational weight gain (GWG) and a lower risk of adverse perinatal outcomes [1,2]. Despite the benefits of physical activity, only 32% of pregnant women achieve the recommended amount of at least 150 minutes of moderate physical activity per week throughout pregnancy [3]. As such, pregnant women spend more than 50% of their time being sedentary [4], which is linked to higher GWG, gestational hypertension, and infant macrosomia [4]. This imbalance of physical activity and sedentary behaviors may contribute to inappropriate GWG. As only one-third of pregnant women gain an appropriate amount of weight [5], pregnancy is a critical period for changes to physical activity, which has known benefits for body weight management.

The US Preventive Services Task Force issued a grade B recommendation for using multicomponent behavioral interventions in pregnancy to limit excess weight gain. The review suggested that high-intensity counseling interventions delivered in-person or remotely, such as our SmartMoms pilot intervention [6], can impact maternal outcomes [7]. Our initial SmartMoms trial delivered a multicomponent behavioral intervention either remotely via mobile phone or in person and effectively reduced the proportion of women exceeding the 2009 National Academy of Medicine GWG guidelines compared with Usual Care [8]. The remote delivery method was more cost-effective for both participants and clinics and achieved higher adherence, demonstrating its potential for scalable implementation. Remote eHealth interventions include the use of mobile smartphones and can help overcome barriers to traditional clinic-based treatments such as transportation issues, time restrictions, and access to health care centers [9]. With 97% of US adults owning smartphones, and low-income households most likely to rely solely on them for web-based access [10], smartphones have become a primary gateway to web-based resources. This indicates that smartphone-based interventions can be a powerful tool for reaching underserved populations with limited access to financial and health care resources.

Pregnant women who face economic disadvantages have an increased risk for adverse perinatal outcomes coupled with reduced access to nutritious foods and less opportunities for safe physical activity [11-13]. These disparities are notably prevalent in Louisiana, where 1 in 5 adults (18.5%) experiences poverty [14]. The federally funded Special Supplemental Nutrition Program for Women, Infants, and Children (WIC) aims to reduce nutrition-related health inequalities through the provision of food vouchers and nutrition education to pregnant women and their families. In its 50 years of existence, the WIC program has contributed to reductions in the prevalence of infant mortality, preterm birth, and childhood anemia in addition to improvements in birth weight and maternal diet quality [15,16]. WIC serves 6.7 million people annually [17], yet no prior randomized controlled trials have evaluated targeted behavioral interventions for managing GWG in this setting. Therefore, WIC’s unique infrastructure has an unparalleled ability to deliver a multicomponent lifestyle intervention with the goal of implementing health behavior change in underserved women during pregnancy, a population that is traditionally hard to reach.

Previous prenatal lifestyle interventions have been shown to attenuate GWG in pregnant women [18-20], but the extent to which GWG benefits are attributable to changes in physical activity remains unclear. In a meta-analysis of 23 randomized controlled trials including 4462 pregnant women, GWG was significantly decreased with exercise performed at least 3 times a week for a minimum of 30 minutes, yet none of the included interventions were eHealth [21]. Leveraging the reach of WIC to underserved communities, we codeveloped an eHealth lifestyle intervention with community stakeholders and conducted the first randomized controlled trial aimed at promoting appropriate GWG among underserved women engaged in a federal supplemental nutrition program [22]. The codeveloped multicomponent intervention consisted of daily weighing, self-monitoring of steps with an activity monitor, physical activity counseling (eg, in the form of videos suggesting an exercise of the week using study-provided exercise equipment), behavioral counseling on diet quality, interaction with health coaches, and social support through a private Facebook group [23]. Participants in the Intervention Group gained less total and weekly weight across gestation than the Usual Care Group [22]. Given the known health benefits of engaging in regular physical activity and decreasing sedentary time, the objective of this prespecified secondary analysis was to (1) examine the impact of this pragmatic, multicomponent lifestyle intervention on physical activity and sedentary time, and (2) explore associations between changes in physical activity and sedentary time and GWG.

## Methods

### Study Design

This is a preplanned secondary analysis of a randomized controlled trial in pregnant women enrolled in Louisiana WIC that was designed to test the effectiveness of a remotely delivered behavior modification program to promote appropriate weight gain during pregnancy as recommended by the 2009 National Academy of Medicine (NAM) GWG per week guidelines (Clinical Trial Registration: NCT04028843) [24]. The CONSORT-EHEALTH (Consolidated Standards of Reporting Trials of Electronic and Mobile Health Applications and Online Telehealth) checklist is included in Multimedia Appendix 1. A detailed report describing how the trial outcomes, eligibility criteria, trial design, and intervention content were codeveloped with community stakeholders at the Baton Rouge Community Advisory Board of the Louisiana Clinical and Translational Science Center and a WIC Mothers’ Advisory Group at monthly meetings has been published [22,23]. Briefly, based on collective recommendations, the intervention curriculum was designed to be culturally sensitive, relatable, and aligned with participants’ literacy levels. Recommendations included having weekly health education, social support mechanisms such as closed Facebook groups, and culturally relevant, low-literacy materials delivered in short videos featuring topics such as WIC-related recipes and pregnant women exercising.

Recruitment took place from July 2019 to December 2023 at 31 WIC clinics across Louisiana, selected by size and location. Pregnant women receiving WIC benefits who owned a smartphone with internet access were introduced to the trial in-clinic or via phone or text by the clinic study staff. All marketing materials demonstrated affiliation with Pennington Biomedical Research Center and WIC. Interested participants participated in a phone screening call to receive more information about the study and be assessed for eligibility. If participants wished to proceed, an in-person screening visit was scheduled by the clinic staff to review the study consent form and confirm eligibility. Participants were randomized (1:1, parallel arm) by their assigned health coach after the completion of the first outcome visit to either the Healthy Beginnings mobile eHealth intervention in addition to WIC services (Intervention Group) or the usual WIC Nutrition only (Usual Care Group), with randomization stratified by BMI (normal weight, overweight, and obesity) and Louisiana Department of Health region. The randomization allocation sequence was concealed to intervention staff prior to group assignment and the trial adhered to a single-blind design with clinic staff and health coaches kept separate. The intervention spanned approximately 24 weeks depending on gestational age at enrollment and gestational age at delivery with participants being randomized between the 10th and 16th weeks of pregnancy and participating until delivery. Outcomes were assessed by blinded clinic staff members at 3 in-person study visits that occurred during early (10.0-16.6 weeks of gestation), mid (24.0-27.6 weeks), and late (35.0-36.6 weeks of gestation) pregnancy. The COVID-19 pandemic caused a 5-month recruitment halt. To meet the December 2023 enrollment deadline, the overall sample size was adjusted downward as previously described [22]. The primary outcome of GWG has been published [22]; the present analysis evaluates the preplanned secondary outcome of changes in physical activity from study visits conducted in early and late pregnancy. At these visits, participants completed the Pregnancy Physical Activity Questionnaire [25] and were administered an accelerometer to wear over 3 to 7 days to assess habitual physical activity and sedentary time.

### Ethical Considerations

The study aims and outcomes were approved and monitored by the institutional review boards at Pennington Biomedical Research Center (2018-039-PBRC) and Louisiana Department of Health, and participants provided written informed consent prior to study procedures. The informed consent specified that every effort would be made to maintain the confidentiality of study records, specifically through deidentifying private information. Participants were compensated up to US $150 for completion of the study or compensated US $25 for each study visit if the study was not completed.

### Participants

Eligible individuals were pregnant with a singleton viable pregnancy, BMI between 18.5 and 40 kg/m^2^ from study-measured weight and height, gestational age of ≥10.0 weeks but ≤16.6 weeks at enrollment, and certified to receive WIC services during the current pregnancy. Exclusion criteria included age of <18 or >40 years; current use of drugs, tobacco or smoking, or alcohol; chronic illness (eg, HIV, cancer, heart disease, and diabetes); hypertension (systolic blood pressure >160 mm Hg or diastolic blood pressure >110 mm Hg); or current mental health or eating disorder [22,23]. Inclusion in this secondary analysis also required that participants had at least 3 days of valid accelerometry data (≥10 hour of awake wear time per day) at either early or late pregnancy time point [26]. In addition, at baseline we collected demographic and socioeconomic information from questionnaires, dietary intake data from the Automated Self-Administered 24-Hour, and used participants’ zip codes to derive environmental (neighborhood) descriptors via the Area Deprivation Index [27] as well as the Maternal Vulnerability Index [28].

### Treatment Groups

In brief, the intervention was an approximately 24-week, evidence-based, intensive mobile eHealth multicomponent behavioral modification program aimed at helping pregnant individuals achieve BMI-specific GWG within the ranges recommended by NAM. Key components of the intervention were daily weighing, recording steps, educational lessons, and health coach contacts. Specifically, the physical activity component of the intervention promoted moderate-intensity physical activity of 150 minutes per week, as recommended during pregnancy. The focus of the physical activity component was to reduce sedentary time through increasing lifestyle activities (eg, reaching 5000-10,000 steps per day), such as taking the stairs instead of the elevator, actively commuting (eg, walking or cycling) whenever possible, and replacing sedentary activities with more active options. Participants were given an intervention tool kit at enrollment that included exercise equipment (ie, yoga mat, resistance bands, and dumbbells), items for meal preparation (ie, measuring cups, reusable water bottle, and lunch boxes), and educational material on healthy behaviors such as healthy recipes using WIC redemption foods.

Participants received a digital scale to self-monitor their body weight daily and an activity tracker (Fitbit) to monitor their daily physical activity data (ie, step count). The Fitbit app and scale web page enabled individuals to self-monitor their behaviors, view their progress, and receive automated feedback. The participant-facing platform for the scale generated a NAM weight gain chart personalized to participants’ weight at baseline that was used to provide automated prescriptive feedback on change in weight. If participants’ weight exceeded the target range, either above or below the predicted GWG, it was classified as “out of the zone.” A stoplight visual was used to display progress, where green indicated that weight was within the predicted range, yellow signaled that weight was approaching the upper or lower limit, and red denoted that the weight was “out of the zone.” In addition, participants received weekly educational content via email in the form of brief 2- to 4-minute videos featuring intervention staff and actors reflective of the study population (topics listed in Multimedia Appendix 2). These videos delivered health information and practical guidance to support behavior change, with an emphasis on dietary intake, physical activity, and stress management.

Participants’ body weight and activity data were automatically and wirelessly transmitted to a clinician dashboard (Virtual Weight Management Suite) in near real time with very little participant or staff burden. Health coaches were able to evaluate whether participants were weighing daily and where the weights were relative to the NAM weight gain chart. Participants received weekly one-on-one virtual check-ins from a health coach via a web-based messaging platform, where they were provided with personalized feedback on physical activity and dietary behaviors based on their weight gain chart. Participants could also initiate messages to their health coach through the platform at any time. The weekly check-ins allowed health coaches to provide education on physical activity, diet, and other health behaviors discussed in the weekly educational content and to provide personalized feedback when participant trends indicated insufficient physical activity (eg, 5000 steps per day) or weight gain outside the recommended NAM guidelines.

Participants interacted with one another in a private, closed Facebook group designed to provide social support and foster community engagement throughout the intervention. Health coaches regularly posted brief educational content including health-related discussion prompts, recipes featuring WIC foods, and weekly “exercise of the week” videos (topics listed in Multimedia Appendix 3). Exercise videos featured the exercise equipment that was provided to the participants. Participants were encouraged to respond to the study Facebook posts, share their experiences, and support one another through comments and reactions. Interventionists, including health coaches and research assistants, actively facilitated engagement by initiating discussion topics, responding to participant comments, and prompting dialogue around weekly themes related to healthy pregnancy, nutrition, physical activity, and postpartum care. All posts and comments were monitored by study staff to ensure that discussions remained appropriate and primarily focused on intervention-related topics, while allowing space for supportive peer-to-peer interaction that extended beyond the structured curriculum.

The Usual Care Group received the same exercise equipment and dietary tools as the Intervention Group, and they received general health information via email that was not related to weight management (Multimedia Appendix 2). Participants were included in a separate closed Facebook group in which weekly posts related to general pregnancy health were shared and were contacted monthly by a health coach via phone or text message for retention.

Both groups received all aspects of the Louisiana WIC program. However, health professionals at WIC did not aid with the mobile eHealth intervention offered by the study. The mobile eHealth intervention was provided in addition to the standard of care from WIC. Study health coaches had college degrees in nutrition, kinesiology, or psychology, and followed a treatment manual under the supervision of trial investigators. Intervention fidelity was maintained through the use of a study-specific intervention manual and weekly case conferences. Quality assurance methods for the study included a data safety and monitoring plan, double data entry for outcomes, chart auditing, and regular checks for missing data.

### Procedures

#### Movement Behaviors

##### Movement Duration

Physical activity was assessed for up to 7 days in early and late pregnancy using the ActiGraph GT3X+ accelerometer (ActigGraph, LLC, Pensacola, FL, USA). Participants were asked to wear the accelerometer on their nondominant wrist [29,30] for 24 hours per day including overnight and while bathing. The monitors were initialized to record at a 50-Hz sampling rate, the highest sampling rate possible for an up to 7-day monitoring period.

The data were downloaded using the ActiLife software (version 6.5.2; ActiGraph, LLC) and the GENEActiv personal computer software (version 2.2; ActivInsights Ltd) and then processed with the GGIR algorithm in R (R Foundation for Statistical Computing) [31]. The GGIR Lite algorithm was used to detect non–wear time. GGIR was used to determine physical activity and sedentary time duration. The Euclidian Norm Minus One (ENMO) value calculated by GGIR as a proxy for energy expenditure was used to classify level of activity (inactive [<44.8 mg], light [44.8 mg to ≤ ENMO <100.6 mg], moderate [100.6 mg to ≤ ENMO < 428.8 mg], and vigorous [>428.8 mg]) and the time spent in each activity [32-36]. To avoid capturing random wrist movements, only activity bouts of ≥1 minute where 80% of the activity were classified as moderate to vigorous physical activity (MVPA) were included to capture physical activity [36,37].

Variables were summarized for each participant across valid days and each time point as counts or nonweighted averages. Data collected from the accelerometer relevant for this analysis included inactive time (minutes per day), light activity (minutes per day), moderate activity (minutes per day), vigorous activity (minutes per day), MVPA (minutes per day), and number of 1- to 5-minute MVPA bouts per day. Inactive time was renamed sedentary time to align with consensus for reporting sedentary measures [38].

##### Movement Context

Participant-reported physical activity and sedentary time was collected with the Pregnancy Physical Activity Questionnaire: a validated questionnaire that evaluates time spent on 33 activities categorized into household or caregiving (16 activities), occupational activities (5 activities), sports or exercise (9 activities), and transportation (3 activities) [25]. Participants recalled the time spent on each activity during the current trimester. Response options varied by activity, which included activities performed while not at work or going places (0-3+ hours per day), activities completed for fun or exercise (0-3+ hours per week), and activities performed at work (0-6+ hours per day). Two open-ended questions allowed participants to provide any additional activities they participated in for fun or for exercise during the week. The time spent in each activity was multiplied by its intensity (sedentary, light, moderate, or vigorous) to compute at a measure of average weekly energy expenditure (metabolic equivalent task [MET]–hours per week where 1 MET is the metabolic equivalent of the energy expended at rest) attributable to each activity. These values were then summed to derive the weekly total activity sum. The energy expended was also calculated according to each activity domain and each intensity level (sedentary [<2.0 METs], light [2.0 ≤ activity <3.0 METs], moderate [3.0 ≤ activity ≤6.0 METs], or vigorous [>6.0 METs]) [39].

#### Gestational Weight Gain

Weight was measured in light clothing and without shoes using a digital scale at study visits. Study-observed GWG (kilogram) was calculated as the difference between the early and late pregnancy visit weights. GWG per week (kilogram per week) was calculated as study-observed GWG divided by the number of weeks between visits. For participants who missed their late pregnancy visit (eg, due to an early delivery) but had an intermediary midpregnancy weight data point, weekly GWG was calculated analogously based on weight assessed at that time point. GWG guideline attainment was based on weekly GWG and defined as meeting the 2009 NAM weight gain recommendations in the second and third trimesters for pregnant women with normal weight (0.35-0.50 kg per week), overweight (0.23-0.33 kg per week), or obesity (0.17-0.27 kg per week) [22,40].

### Statistical Analysis

Data are presented as mean (SD) or percentages for continuous or categorical variables, respectively. For all outcomes, *P* values of <.05 were considered to indicate statistical significance. Analyses were performed using R (version 4.3.2; R Foundation for Statistical Computing).

Physical activity data were analyzed using a linear mixed model including fixed effects for time (early and late pregnancy) and group (Intervention Group and Usual Care Group) as well as the interaction, and a random effect for participant. The model was adjusted for BMI category (normal weight, overweight, and obesity) at randomization and all individuals with available data at either time point were included in the model but missing data were not imputed [41]. Model-derived estimated marginal means alongside corresponding 95% CIs as well as within- and between-group effect estimates with associated CIs and *P* values are reported. The main effect of interest is the between-group effect estimate. Furthermore, the treatment heterogeneity across the different BMI categories was explored via a 3-way interaction including time, group, and BMI category, with custom comparisons exploring intervention effects on physical activity within and between BMI categories.

To examine the effect of changes in physical activity on GWG outcomes (study-observed GWG, weekly GWG, and GWG guideline attainment), we used data from all participants collapsed across Intervention Groups (ie, independent of randomization assignment). These analyses used a linear model including a fixed effect for the change across the intervention period in both MVPA and inactivity (adjusting for each other in the same model) as measured via ActiGraph and adjustments for BMI category at randomization, parity (categorically defined as nulliparous vs nonnulliparous), and gestational age at enrollment. Effects are presented as regression coefficients (study-observed GWG and weekly GWG) or odds ratios (GWG guideline attainment) for each predictor with 95% CIs and corresponding *P* values.

## Results

### Participants

**Figure 1:**
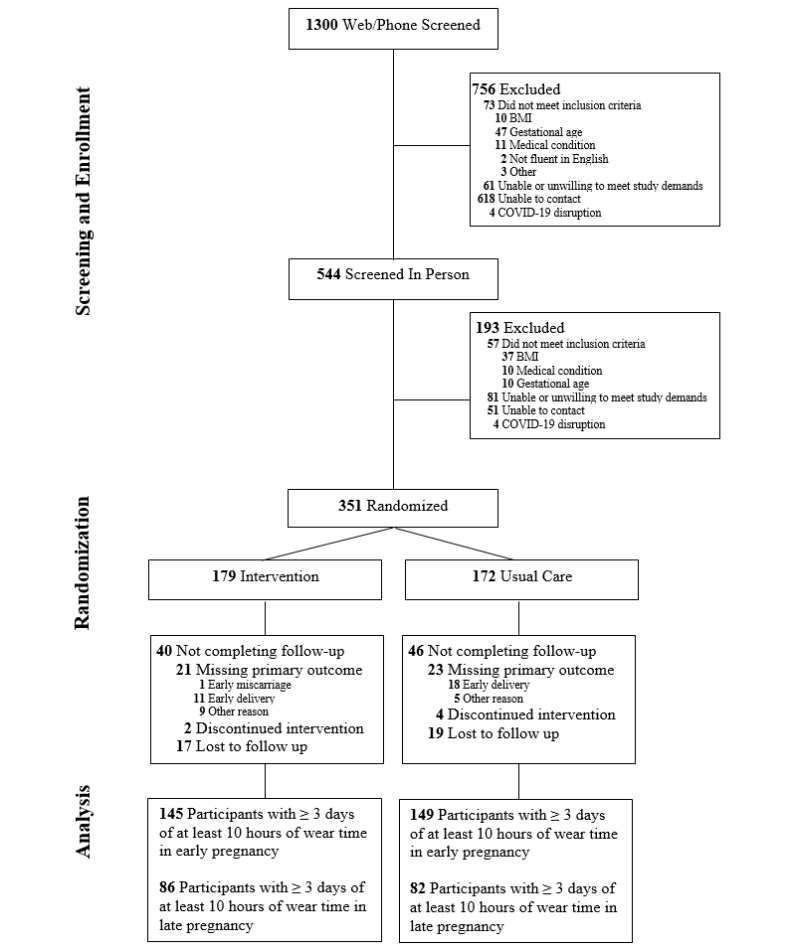
CONSORT (Consolidated Standards of Reporting Trials) diagram.

The CONSORT (Consolidated Standards of Reporting Trials) diagram is included in Figure 1. Participants mostly identified as non–Hispanic Black (201/351, 57%) and were aged 27.0 (SD 6.0) years, 15.2 (SD 1.6) weeks of gestation, with pregnancy weight at enrollment of 75.1 (SD 16.9) kg, and corresponding BMI of 28.5 (SD 5.8) kg/m^2^ (Table 1). At delivery, incidence of gestational diabetes (Intervention [n=13/179] and Usual Care [n=13/172]), gestational hypertension (Intervention [n=26/179] and Usual Care [n=29/172]), and medically indicated cesarean (Intervention [n=36/179] and Usual Care [n=37/172]) was low. Of the 351 enrolled participants, 25 (7%) participants were excluded from this analysis due to missing accelerometry data at both time points (ie, neither early nor late pregnancy accelerometry was available), and an additional 13 (4%) were excluded due to an inadequate number (ie, 3) of valid days at either time point. Overall, 89% (313/351) of participants had ≥3 valid days for at least 1 time point, with 84% (294/351) and 48% (168/351) having at least 3 valid days at the early and late pregnancy time points, respectively.

**Table 1:** Baseline characteristics for participants by randomization assignment^a^.

Demographic characteristics				Overall (N=351)		Intervention (N=179)		Usual Care (N=172)
Gestational age at randomization (weeks), mean (SD)				15.2 (1.6)		15.2 (1.6)		15.1 (1.6)
Maternal age (years), mean (SD)				27.0 (6.0)		27.0 (6.0)		28.0 (6.0)
Body weight (kg), mean (SD)				75.1 (16.9)		74.8 (17.5)		75.4 (16.4)
**BMI (kg/m^2^), mean (SD)**				28.5 (5.8)		28.6 (5.9)		28.5 (5.7)
		Normal weight, n (%)	114 (33)		58 (32)		56 (33)	
		Overweight weight, n (%)	99 (28)		51 (29)		48 (28)	
		Obesity, n (%)	138 (39)		70 (39)		39 (39)	
**Race or ethnicity, n (%)**								
		Hispanic	23 (7)		10 (6)		13 (8)	
		Non-Hispanic White	109 (31)		55 (31)		54 (31)	
		Non-Hispanic Black	201 (57)		106 (59)		95 (55)	
		Multiracial/Other	18 (5)		8 (4)		10 (6)	
**Education level, n (%)**								
		College education	221 (63)		110 (61)		111 (65)	
		No college education	130 (37)		69 (39)		61 (35)	
**Marital status, n (%)**								
		Married or living with significant other	200 (57)		104 (58)		96 (56)	
		Not married	151 (43)		75 (42)		76 (44)	
**Dietary intake** **, mean (SD)**								
	Calories (kcal/day)		1965.9 (803.9)		1992.0 (785.6)		1939.1 (823.8)	
	Carbohydrates (grams)		230.3 (105.0)		235.2 (103.1)		225.2 (107.1)	
	Protein (grams)		81.7 (39.9)		81.1 (37.1)		82.3 (42.6)	
	Fat (grams)		81.5 (37.4)		82.7 (36.6)		80.4 (36.6)	
**Environmental descriptors**								
		Area deprivation index (national percentile), mean (SD)	68.0 (18.7)		68.4 (18.6)		67.6 (18.8)	
		Maternal vulnerability index ( arbitrary units), mean (SD)	74.7 (12.8)		75.2 (12.7)		74.1 (12.9)	
		Nulliparous, n (%)	150 (43)		84 (48)		66 (38)	
**ActiGraph accelerometer wear, N**				249^b^		145^b^		149^b^
		Total valid days, mean (SD)	5.5 (1.2)		5.5 (1.4)		5.5 (1.0)	
		Weekdays, mean (SD)	3.6 (1.0)		3.7 (1.1)		3.5 (0.9)	
		Weekends, mean (SD)	1.9 (0.5)		1.8 (0.6)		1.9 (0.3)	
		≥3 valid days, %	84		81		87	

^a^Data are presented as mean (SD) or % (n).

^b^Data presented for participants with ≥3 days of at least 10 hours of wear time in early pregnancy. Higher values for area deprivation index and maternal vulnerability index indicate higher disadvantage and vulnerability, respectively; national area deprivation index (0-100) and maternal vulnerability index (0-100).

### Intervention Engagement

Approximately two-thirds of the participants engaged with self-monitoring behaviors on 3 or more days per week (Multimedia Appendix 4). On average, participants in the Intervention Group recorded their steps 3.6 (SD 2.7) days per week. Fitbit data were available from 83% (148/179) of participants in the Intervention Group. Of the 149 participants, more than half (83/149, 56%) recorded their steps at least 3 days per week, with more than 40% (60/149) recording their step count on more than 5 days per week on average. On average, participants achieved their step goal of 5000 and 10,000 steps per day on 39% (SD 26%) and 8% (SD 13%), respectively, of the days that they recorded steps. Participants took an average of 4560 (SD 2168) steps per day across the intervention period, which was consistent between the second (mean 4785 steps per day, SD 2293) and third (mean 4602 steps per day, SD 2231) trimesters.

### Accelerometer Movement Duration

#### Baseline

Participants across both groups spent more than 10 hours per day of their waking time in sedentary activities, with an additional approximately 3 hours and 1.25 hours per day spent doing light and moderate activity, respectively (Table 2). The proportion of time spent in each movement behavior was similar across BMI categories (Multimedia Appendix 5). Almost all participants met the physical activity guideline recommendations of at least 150 minutes per week of MVPA per week in early (~22.5 minutes per day; 142/145, 98% in the Intervention Group and 146/149, 98% in the Usual Care Group) and late (79/86, 92% in the Intervention Group; 80/82, 98% in the Usual Care Group) pregnancy (Multimedia Appendix 6).

**Table 2:** Actigraph accelerometry–measured physical activity levels during early and late pregnancy per randomization assignment^a^.

	Intervention				Usual Care				Between-group *P* value
	Early pregnancy (N=145)	Late pregnancy (N=86)	Within-group change	Early pregnancy (N=149)		Late pregnancy (N=82)	Within-group change		
Sedentary time (minutes per day)	698 (682 to 715)	761 (741 to 781)	62 (42 to 83)^b^	686 (670 to 702)		738 (716 to 758)	52 (31 to 72)^b^	.47	
Light activity (minutes per day)	182 (174 to 190)	184 (174 to 194)	2 (–8 to 13)	189 (182 to 197)		183 (173 to 193)	–7 (–17 to 3)	.21	
Moderate activity (minutes per day)	74 (69 to 80)	61 (54 to 69)	–13 (–20 to –6)^b^	81 (76 to 87)		72 (64 to 79)	–10 (–17 to –3)^b^	.57	
Vigorous activity (minutes per day)	2 (1 to 3)	1 (0 to 2)	–1 (–2 to 0)	2 (1 to 3)		2 (1 to 3)	0 (–1 to 1)	.35	
MVPA^c^ (minutes per day)	77 (71 to 83)	62 (55 to 70)	–14 (–21 to –7)^b^	83 (77 to 89)		73 (66 to 80)	–10 (–18 to –3)^b^	.45	
MVPA 1- to 5-minute bouts per day	15 (13 to 17)	11 (8 to 13)	–4 (–7 to –2)^b^	18 (16 to 20)		13 (11 to 16)	–4 (–7 to –2)	.91	

^a^Data presented as estimated marginal means alongside corresponding 95% CIs with within- and between-group effect estimates and *P* values derived from a linear mixed model including fixed effects for time and group as well as the interaction, and a random effect for participant. Model adjusted for BMI category at enrollment.

^b^Within-group change (*P*<.05).

^c^MVPA: moderate to vigorous physical activity.

#### Change Within Groups

**Figure 2:**
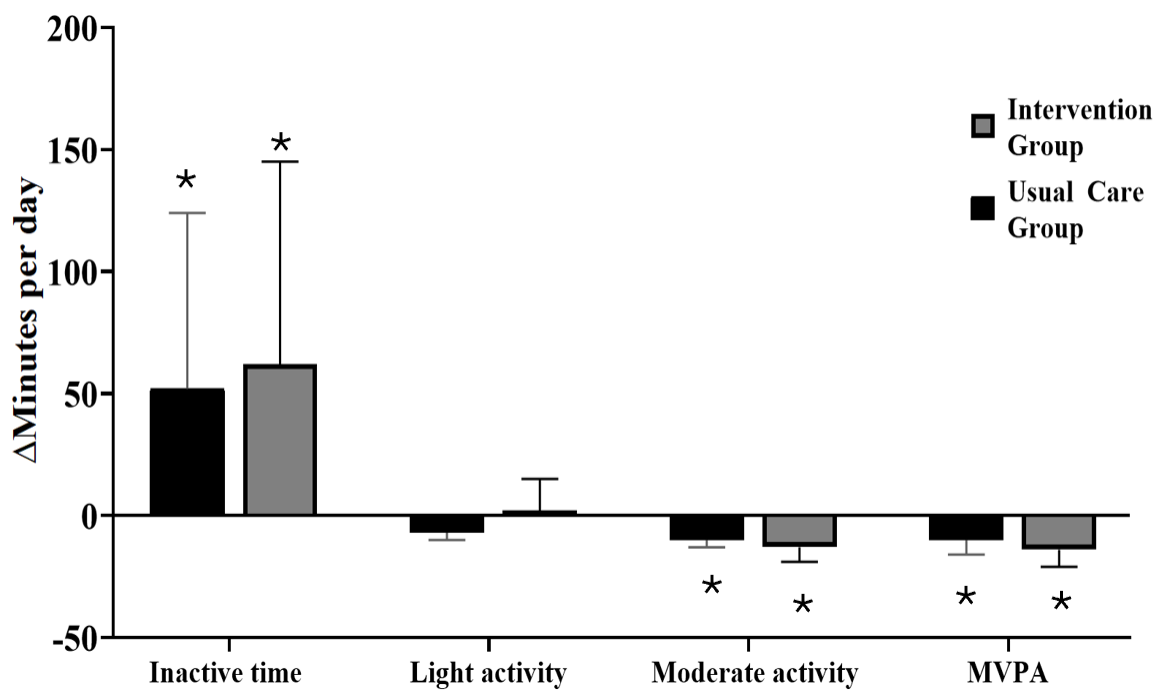
A comparison of physical activity changes from early to late pregnancy assessed via accelerometry between the Intervention and Usual Care Groups. *Significant within-group change from early to late pregnancy (*P*<.05). There were no significant differences between groups for these outcomes. MVPA: moderate to vigorous physical activity.

Participants in the Intervention Group significantly increased sedentary time (adjusted effect estimate 62, 95% CI 42-83 minutes per day, *P*<.001) and significantly decreased moderate activity (–13, 95% CI –20 to –6 minutes per day, *P*<.001), total MVPA (–14, 95% CI –21 to –7 minutes per day, *P*<.001), and MVPA in 1- to 5-minute bouts (–4, 95% CI –7 to –2 bouts per day, *P*<.001) from early to late pregnancy (Table 2 and Figure 2). Similarly, the Usual Care Group significantly increased inactivity time (52, 95% CI 31-72 minutes per day, *P*<.001) and significantly decreased moderate activity (–10, 95% CI –17 to –3 minutes per day, *P*=.01), total MVPA (–10, 95% CI –18 to –3 minutes per day, *P*=.01), and 1- to 5-minute MVPA bouts (–4, 95% CI –7 to –2 bouts per day, *P*<.001; Table 2 and Figure 2).

#### Change Between Groups

From early to late pregnancy*,* there were no significant differences between the Intervention and Usual Care Groups for the change in any physical activity measure (Table 2). When examining between-group changes separately by BMI categories, there was an intervention effect within obesity for sedentary time (48, 95% CI 1-95, *P*=.04), suggesting a greater increase in sedentary time in the Intervention Group in individuals with obesity. There were no between-group differences within or between BMI categories in physical activity measures (Multimedia Appendix 5).

### Participant Self-Reported Movement Context

#### Baseline

At baseline (ie, in early pregnancy), participants in both groups reported approximately 300 MET-hours per week of total activity, which mainly comprised 120-130 MET-hours per week of both light and moderate activity with very little vigorous activity, and approximately 45 MET-hour per week of sedentary activity (Table 3). Most of the activity was reported to result from within household and occupational contexts, with limited activity arising from transportation (<30 MET-hours per week) or sports (~15 MET-hours per week).

**Table 3:** Self-reported physical activity during early and late pregnancy per randomization assignment^a^.

		Intervention										Usual Care										Between-group *P* value		
		Early pregnancy (N=179)			Late pregnancy (N=140)			Within-group change			Early pregnancy (N=172)				Late pregnancy (N=125)			Within-group change						
**Movement behaviors (MET^b^-hour per week)**																								
	Total activity			301 (277 to 324)			269 (243 to 295)			–32 (–57 to –6)^c^				307 (283 to 332)			269 (241 to 296)			–39 (–65 to –12)^c^				.71
	Sedentary time			48 (44 to 52)			44 (40 to 48)			–4 (–9 to 0)^c^				43 (38 to 47)			43 (39 to 48)			1 (–4 to 5)				.10
	Light activity			123 (113 to 134)			120 (109 to 131)			–3 (–14 to 8)				129 (119 to 140)			124 (113 to 136)			–5 (–16 to 7)				.86
	Moderate activity			126 (110 to 141)			101 (84 to 118)			–25 (–43 to –7)^c^				132 (117 to 148)			99 (81 to 116)			–34 (–53 to –15)^c^				.49
	Vigorous activity			3 (3 to 4)			3 (3 to 4)			0 (–1 to 1)				3 (3 to 4)			3 (2 to 4)			–1 (–1 to 0)				.26
**Contextual behaviors** **(MET-hour per week)**																								
	Household activity		108 (96 to 120)			98 (85 to 112)			–10 (–21 to 2)				112 (100 to 125)			103 (89 to 117)			–9 (–22 to 3)				.98	
	Occupation activity		96 (80 to 112)			77 (59 to 94)			–19 (–38 to 0)^c^				105 (89 to 121)			76 (58 to 95)			–29 (–48 to –9)^c^				.47	
	Sports activity		14 (12 to 16)			16 (15 to 18)			2 (0 to 4)^c^				15 (13 to 16)			13 (11 to 14)			–2 (–4 to 0)^c^				.002	
	Transportation activity		28 (23 to 32)			27 (22 to 32)			–1 (–7 to 5)				26 (21 to 31)			26 (21 to 31)			0 (–6 to 6)				.81	
	Sedentary time		55 (51 to 60)			51 (46 to 56)			–4 (–9 to 1)				49 (45 to 54)			51 (45 to 56)			1 (–4 to 7)				.14	

^a^Data presented as estimated marginal means alongside corresponding 95% CIs with within- and between-group effect estimates and *P* values derived from a linear mixed model including fixed effects for time and group as well as the interaction thereof, and a random effect for participant. Model adjusted for BMI category at enrollment.

^b^MET: metabolic equivalent of the energy expended at rest.

^c^Within-group change (*P*<.05).

#### Change Within Groups

From early to late pregnancy, the Intervention Group reported a significant decrease in total activity (–32, 95% CI –57 to –6 MET-hours per week, *P*=.01), moderate activity (–25, 95% CI –43 to –7 MET-hours per week, *P*=.01), occupational activity (–19, 95% CI –38 to 0 MET-hours per week, *P*=.04), and sedentary time (–4, 95% CI –9 to 0 MET-hours per week, *P*=05). Similarly, within the Usual Care Group, participants reported significantly decreased total activity (–39, 95% CI –65 to –12 MET-hours per week, *P*=.01), moderate activity (–34, 95% CI –53 to –15 MET-hours per week, *P*<.001), and occupational activity (–29, 95% CI –48 to –9 MET-hours per week, *P*=.004) from early to late pregnancy (Table 3).

#### Change Between Groups

From early to late pregnancy*,* the Intervention Group reported an increase in sports participation (2, 95% CI 0-4 MET-hours per week, *P*=.02) whereas the Usual Care Group reported a decrease in sports participation (–2, 95% CI –4 to 0 MET-hours per week, *P*=.04), resulting in a statistically significant between-group difference (4, 95% CI 2-7 MET-hours per week, *P*=.002; Table 3). There were no other statistically significant differences between groups over time in self-reported physical activity or sedentary activities.

### Movement Duration and GWG

As there were no statistically significant or clinically meaningful differences in physical activity changes between the Intervention and Usual Care Groups, we combined the groups to evaluate the effect of movement duration on GWG. Changes in sedentary time (adjusted β=4, 95% CI –4 to 12 g; *P*=.31) or MVPA (–7, –32 to 18 g; *P*=.57) were not related to study-observed GWG. Similarly, inactivity time (0, 95% CI 0-1 g per week; *P*=.30) or MVPA (0, 95% CI –1 to 1 g per week; *P*=.49) did not predict weekly GWG. Furthermore, there was no relationship between the change in MVPA and GWG guideline attainment (adjusted odds ratio 0.99, 95% CI 0.97-1.01; *P*=.58) or between the change in inactivity and GWG guideline attainment (1.00, 95% CI 0.99-1.01; *P*=.91).

## Discussion

### Principal Results

This study assessed the impact of the first mobile eHealth behavior modification program adapted for pregnant individuals in Louisiana WIC on movement behaviors measured by accelerometry and participant self-report, and its impact on GWG. Almost all participants met the physical activity guideline recommendations of at least 150 minutes per week of MVPA at baseline in early pregnancy (98% in the Intervention Group and 98% in the Usual Care Group). There were no significant between-group differences in accelerometry-derived physical activity over time, but within both groups, physical activity outcomes measured by accelerometry decreased from early to late pregnancy. In agreement, sedentary time significantly increased similarly across gestation within both groups. In contrast, the Intervention Group reported an increase in sport participation and a significant decrease in sedentary time from early to late pregnancy. Changes in physical activity or sedentary time did not moderate total GWG or weekly GWG. As this is the first randomized controlled trial to test a GWG intervention within the WIC program, there is little existing literature available for direct comparison of our findings. More opportunities to sustain an adequate movement behavior across gestation in pregnant women facing economic disadvantages are needed.

### Comparison With Prior Work

In this study, both the Intervention and Usual Care Groups demonstrated a decrease in moderate physical activity and total MVPA from early to late pregnancy (range –14 to –10 minutes per day) and self-reported decreased moderate physical activity and total activity (–25 to –39 MET-hours per week). These within-group trends were consistent within each BMI category. Our findings are consistent with another study that assessed how a lifestyle intervention based on a brochure (passive) or on in-person education sessions (active) compared with a control group in White pregnant women with obesity (n=195) affected physical activity. The study demonstrated a decrease in physical activity from early to late pregnancy within each group (passive, active, or control) but lacked differences between groups showing that physical activity decreased for all study participants across pregnancy [42]. In addition, we found that sedentary time measured objectively with accelerometry increased across pregnancy and in both groups (+52 minutes per day for the Usual Care Group and +62 minutes per day for the Intervention Group), while self-reported sedentary time did not change across pregnancy (–4 MET-hour per week for Intervention Group and 1 MET-hour per week for the Usual Care Group). Our values of sedentary time align with previous investigations in pregnant women (range 420-760 minutes per day) and suggest that pregnant women increase their sedentary time during pregnancy regardless of socioeconomic status [1,4,43].

We used a consultative research approach to inform the physical activity component of the intervention. With the goal of better meeting the needs of underserved women facing economic disadvantages [23], the feedback from the WIC community stakeholders resulted in the provision of a Fitbit, development of exercise aids (photographs and videos) featuring pregnant women, and the provision of exercise equipment that could be used at home [23]. Even so, the provision of self-monitoring tools and a passive prescription of physical activity did not increase physical activity across pregnancy in this unique population. However, the Intervention Group self-reported an increase in sport participation whereas the Usual Care Group reported a decrease. Previous trials in various settings have evaluated the effects of MVPA interventions lasting 8-30 weeks, varying in intensity from high-intensity, supervised in-person sessions (active interventions) to low-intensity approaches, such as encouraging 10,000 steps per day with self-monitoring (passive interventions) [1,7]. These studies demonstrate that the relationship between the intensity of the intervention and the effects on physical activity is closely related. The generally null results of the current trial suggest that without supervised exercise sessions, mobile eHealth interventions promoting the national physical activity guidelines and providing resources may reduce total GWG [22] but are unlikely to significantly impact physical activity levels during pregnancy in underserved, hard-to-reach populations often excluded from biomedical research, even when that intervention was codeveloped with stakeholders in the community. Together, this study and others in physically inactive pregnant women with overweight or obesity suggest that more intensive interventions are needed to maintain physical activity across pregnancy [44].

The present prespecified analyses did not observe a relationship between changes in movement duration and GWG. While our mobile eHealth intervention included a physical activity component, the change in physical activity was not the primary outcome of the intervention. A systematic evaluation of the existing evidence across 18 studies (including 4 eHealth studies) with 1934 pregnant women having various BMIs and high socioeconomic status found that physical activity interventions in pregnant women result in a decrease of approximately 0.69 (95% CI –1.30 to –0.08) kg (*P*=.03) in total GWG compared with comparator groups [45]. Possible reasons we did not see an effect of change in MVPA or movement behaviors on GWG in this study may be due to greater physical demands from home chores, family responsibilities, and physically intensive jobs, rather than structured exercise, in this population [46]. In addition, other lifestyle interventions in socioeconomically disadvantaged individuals have cited barriers to success such as unsafe neighborhoods limiting the ability to exercise, time demands of occupation, and lack of access to exercise equipment [46]. Our participants lived in neighborhoods or geographic areas that experienced a greater degree of socioeconomic disadvantage and lived in areas with a greater risk of poor maternal health outcomes compared with the national average. To give additional support to the participants, our intervention provided items to assist with the utilization of their existing physical environment (eg, equipment and home-based exercise videos) and social environment (eg, Facebook group). However, larger policy, systems, and large-scale environmental factors may be needed to maintain or increase physical activity in economically disadvantaged populations, particularly in those who are enrolled federal programs such as WIC [47]. Despite the uncertain impact of physical activity changes on GWG in the present trial, previous trials in different socioeconomic settings have demonstrated that physical activity in the perinatal period provides many maternal health benefits, including improved glucose control, reduced systemic inflammation, and reduced risks for preeclampsia and gestational hypertension, even without changing GWG [1,48,49]. While these measures were beyond the scope of the present trial, rates of gestational diabetes and gestational hypertension upon delivery were low in our sample.

### Strengths and Limitations

This study is the largest trial of its kind conducted in a federal supplemental nutrition program, marking a significant and novel contribution to maternal health research. Strengths of this prespecified secondary outcome analysis of changes in movement behaviors in the context of an innovative mobile eHealth multicomponent lifestyle intervention included multiple methods for assessing movement behaviors across the state of Louisiana in an economically disadvantaged population. We obtained multiple days of objective data in a population that is often hard to reach. Our rigorous randomized controlled trial included masked clinical staff for primary outcomes, thereby minimizing bias outcome assessment. Furthermore, the intervention was developed with input from stakeholders at the Baton Rouge Community Advisory Board of the Louisiana Clinical and Translational Science Center and a WIC Mothers’ Advisory Group to directly address the needs of pregnant women in WIC and provide targeted assistance tailored to this unique population. Yet, our study has limitations related to study conduct, movement behavior assessment, and varying interaction levels with the intervention. First, our study was conducted during the COVID-19 pandemic, which may have negatively influenced physical activity habits in our population. Second, the self-report physical activity method may be biased based on participant perception of their daily activities and their health literacy. However, the use of the ActiGraph as a device measure of physical activity strengthens the current methodology, and using self-report and device-based measures is considered the best practice for holistic evaluation of physical activity. Even so, there is no consensus on processing data from wrist-worn devices, definition of valid days, or use of cut points or threshold considerations, although we used methods and analysis software aligning with previous research [50].

### Conclusions

In summary, this prespecified secondary analysis of the first mobile eHealth multicomponent behavioral lifestyle intervention in Louisiana WIC found that including guidance to increase physical activity toward national guidelines did not meaningfully impact physical activity outcomes in pregnant women. Our results demonstrate that physical activity decreased from early to late pregnancy across both Intervention Groups and without effect on GWG. Interestingly, self-reported participation in sports slightly increased in the Intervention Group, suggesting that participants may have perceived themselves to participate in more activities outside of home care, work, and transportation. Our study provides empirical data in a population of pregnant women who face economic disadvantages and are engaged in a federal supplemental nutrition program, thereby provides a foundation for understanding the importance of leveraging multilevel interventions in federal assistantship programs to improve movement behaviors in pregnancy. While our physical activity component was codeveloped with our target population, it did not appear to be intensive enough to support positive physical activity changes in the Intervention Group compared with Usual Care Group.

## Appendix

Multimedia Appendix 1 CONSORT-eHEALTH checklist (V 1.6.1).

Multimedia Appendix 2 Weekly content for one-on-one coaching for the intervention group and monthly one-on-one coaching for the usual care group.

Multimedia Appendix 3 Planned content for Facebook posts over the intervention period.

Multimedia Appendix 4 Intervention engagement metrics.

Multimedia Appendix 5 Device-measured physical activity levels during early and late pregnancy per randomization assignment and BMI category.

Multimedia Appendix 6 A waterfall plot demonstrating the change in moderate to vigorous physical activity (minutes per day) from baseline to the end of pregnancy in the intervention group (gray) and usual care group (black). MVPA: moderate to vigorous physical activity.

## Abbreviations

CONSORT: Consolidated Standards of Reporting Trials
CONSORT-EHEALTH: Consolidated Standards of Reporting Trials of Electronic and Mobile HEalth Applications and onLine TeleHealth
ENMO: Euclidian Norm Minus One
GWG: gestational weight gain
MVPA: moderate to vigorous physical activity
NAM: National Academy of Medicine
WIC: Women, Infants, and Children

## Data Availability

The datasets generated or analyzed during this study are available from the corresponding author on reasonable request.

## References

[1] Gascoigne EL, Webster CM, Honart AW, Wang P, Smith-Ryan A, Manuck TA (2023). Physical activity and pregnancy outcomes: an expert review. Am J Obstet Gynecol MFM.

[2] Ribeiro MM, Andrade A, Nunes I (2022). Physical exercise in pregnancy: benefits, risks and prescription. J Perinat Med.

[3] Ruifrok AE, Althuizen E, Oostdam N, van Mechelen W, Mol BW, de Groot CJM, van Poppel MNM (2014). The relationship of objectively measured physical activity and sedentary behaviour with gestational weight gain and birth weight. J Pregnancy.

[4] Fazzi C, Saunders DH, Linton K, Norman JE, Reynolds RM (2017). Sedentary behaviours during pregnancy: a systematic review. Int J Behav Nutr Phys Act.

[5] Deputy NP, Sharma AJ, Kim SY, Hinkle SN (2015). Prevalence and characteristics associated with gestational weight gain adequacy. Obstet Gynecol.

[6] Altazan AD, Redman LM, Burton JH, Beyl RA, Cain LE, Sutton EF, Martin CK (2019). Mood and quality of life changes in pregnancy and postpartum and the effect of a behavioral intervention targeting excess gestational weight gain in women with overweight and obesity: a parallel-arm randomized controlled pilot trial. BMC Pregnancy Childbirth.

[7] Davidson KW, Barry MJ, Mangione CM, Cabana M, Caughey AB, Davis EM, Donahue KE, Doubeni CA, Krist AH, Kubik M, Li L, Ogedegbe G, Pbert L, Silverstein M, Simon M, Stevermer J, Tseng CW, Wong JB, US Preventive Services Task Force (2021). Behavioral counseling interventions for healthy weight and weight gain in pregnancy: US preventive services task force recommendation statement. JAMA.

[8] Redman LM, Gilmore LA, Breaux J, Thomas DM, Elkind-Hirsch K, Stewart T, Hsia DS, Burton J, Apolzan JW, Cain LE, Altazan AD, Ragusa S, Brady H, Davis A, Tilford JM, Sutton EF, Martin CK (2017). Effectiveness of smartMoms, a novel eHealth intervention for management of gestational weight gain: randomized controlled pilot trial. JMIR Mhealth Uhealth.

[9] Wilson J, Heinsch M, Betts D, Booth D, Kay-Lambkin F (2021). Barriers and facilitators to the use of e-health by older adults: a scoping review. BMC Public Health.

[10] (2023). Mobile fact sheet. Pew Research Center.

[11] Kindratt TB, Moza J, Rethorst CD, Liao Y (2024). How do people spend their day? Sociodemographic disparities in 24-hour movement guideline adherence among US adults using 2017-2020 NHANES Data. J Racial Ethn Health Disparities.

[12] Kracht CL, Katzmarzyk PT, Staiano AE (2021). Household chaos, maternal stress, and maternal health behaviors in the United States during the COVID-19 outbreak. Womens Health (Lond).

[13] Sun J, Piernicka M, Worska A, Szumilewicz A (2023). A socio-ecological model of factors influencing physical activity in pregnant women: a systematic review. Front Public Health.

[14] (2023). Census 2022: poverty, income, and health insurance in Louisiana. Project LB.

[15] Volpp KG, Berkowitz SA, Sharma SV, Anderson CA, Brewer LC, Elkind MS, Gardner CD, Gervis JE, Harrington RA, Herrero M, Lichtenstein AH, McClellan M, Muse J, Roberto CA, Zachariah JP, American Heart Association (2023). Food is medicine: a presidential advisory from the American Heart Association. Circulation.

[16] Venkataramani M, Ogunwole SM, Caulfield LE, Sharma R, Zhang A, Gross SM, Hurley KM, Lerman JL, Bass EB, Bennett WL (2022). Maternal, infant, and child health outcomes associated with the special supplemental nutrition program for women, infants, and children: a systematic review. Ann Intern Med.

[17] (2024). The 2024 state of WIC report: celebrating 50 years of impact and looking toward the future. National WIC Association.

[18] Choi J, Fukuoka Y, Lee JH (2013). The effects of physical activity and physical activity plus diet interventions on body weight in overweight or obese women who are pregnant or in postpartum: a systematic review and meta-analysis of randomized controlled trials. Prev Med.

[19] Fair F, Soltani H (2021). A meta-review of systematic reviews of lifestyle interventions for reducing gestational weight gain in women with overweight or obesity. Obes Rev.

[20] Peaceman AM, Clifton RG, Phelan S, Gallagher D, Evans M, Redman LM, Knowler WC, Joshipura K, Haire-Joshu D, Yanovski SZ, Couch KA, Drews KL, Franks PW, Klein S, Martin CK, Pi-Sunyer X, Thom EA, Van Horn L, Wing RR, Cahill AG, LIFE‐Moms Research Group (2018). Lifestyle interventions limit gestational weight gain in women with overweight or obesity: LIFE-moms prospective meta-analysis. Obesity (Silver Spring).

[21] Wang J, Wen D, Liu X, Liu Y (2019). Impact of exercise on maternal gestational weight gain: an updated meta-analysis of randomized controlled trials. Medicine (Baltimore).

[22] Flanagan E, Falkenhain K, Beyl R, Altazan A, Richard S, Cabre H (2025). Gestational weight gain management in underserved mothers—a state-wide randomized controlled trial in Louisiana WIC. medRxiv. Prepring posted online on 2025.

[23] Flanagan EW, Altazan AD, Comardelle NR, Gilmore LA, Apolzan JW, St Romain J, Hardee JC, Puyau RS, Mayet CL, Beyl RA, Barlow SA, Bounds SS, Olson KN, Kennedy BM, Hsia DS, Redman LM (2020). The design of a randomized clinical trial to evaluate a pragmatic and scalable eHealth intervention for the management of gestational weight gain in low-income women: protocol for the smartMoms in WIC trial. JMIR Res Protoc.

[24] Siega-Riz AM, Bodnar LM, Stotland NE, Stang J (2020). The current understanding of gestational weight gain among women with obesity and the need for future research. NAM Perspect.

[25] Chasan-Taber L, Schmidt MD, Roberts DE, Hosmer D, Markenson G, Freedson PS (2004). Development and validation of a pregnancy physical activity questionnaire. Med Sci Sports Exerc.

[26] Jairo H, Miguele AVR, Florian H, Séverine S, Vincent T (2019). GGIR: a research community-driven open source R package for generating physical activity and sleep outcomes from multi-day raw accelerometer data. J Meas Phys Behav.

[27] Kind AJH, Buckingham WR (2018). Making neighborhood-disadvantage metrics accessible—the neighborhood atlas. N Engl J Med.

[28] The maternal vulnerability. Surgo Ventures.

[29] Hesketh KR, Evenson KR, Stroo M, Clancy SM, Østbye T, Benjamin-Neelon SE (2018). Physical activity and sedentary behavior during pregnancy and postpartum, measured using hip and wrist-worn accelerometers. Prev Med Rep.

[30] Rosenberger ME, Fulton JE, Buman MP, Troiano RP, Grandner MA, Buchner DM, Haskell WL (2019). The 24-hour activity cycle: a new paradigm for physical activity. Med Sci Sports Exerc.

[31] van Hees VT, Gorzelniak L, Dean León EC, Eder M, Pias M, Taherian S, Ekelund U, Renström F, Franks PW, Horsch A, Brage S (2013). Separating movement and gravity components in an acceleration signal and implications for the assessment of human daily physical activity. PLoS One.

[32] Bakrania K, Yates T, Rowlands AV, Esliger DW, Bunnewell S, Sanders J, Davies M, Khunti K, Edwardson CL (2016). Intensity thresholds on raw acceleration data: Euclidean Norm Minus One (ENMO) and mean amplitude deviation (MAD) approaches. PLoS One.

[33] da Silva DF, Mohammad S, Nagpal TS, Souza SCS, Colley RC, Adamo KB (2021). How many valid days are necessary to assess physical activity data from accelerometry during pregnancy?. J Phys Act Health.

[34] (2024). Published cut-points and how to use them in GGIR. R Package Documentation.

[35] Hildebrand M, Hansen BH, van Hees VT, Ekelund U (2017). Evaluation of raw acceleration sedentary thresholds in children and adults. Scand J Med Sci Sports.

[36] Hildebrand M, VAN Hees VT, Hansen BH, Ekelund U (2014). Age group comparability of raw accelerometer output from wrist- and hip-worn monitors. Med Sci Sports Exerc.

[37] da Silva SG, Evenson KR, da Silva ICM, Mendes MA, Domingues MR, da Silveira MF, Wehrmeister FC, Ekelund U, Hallal PC (2018). Correlates of accelerometer-assessed physical activity in pregnancy—the 2015 Pelotas (Brazil) birth cohort study. Scand J Med Sci Sports.

[38] Tremblay MS, Aubert S, Barnes JD, Saunders TJ, Carson V, Latimer-Cheung AE, Chastin SFM, Altenburg TM, Chinapaw MJM, SBRN Terminology Consensus Project Participants (2017). Sedentary Behavior Research Network (SBRN)—terminology consensus project process and outcome. Int J Behav Nutr Phys Act.

[39] Garber CE, Blissmer B, Deschenes MR, Franklin BA, Lamonte MJ, Lee I, Nieman DC, Swain DP, American College of Sports Medicine (2011). American College of Sports Medicine position stand. Quantity and quality of exercise for developing and maintaining cardiorespiratory, musculoskeletal, and neuromotor fitness in apparently healthy adults: guidance for prescribing exercise. Med Sci Sports Exerc.

[40] Rasmussen KM, Yaktine AL (2009). Weight Gain During Pregnancy: Reexamining the Guidelines.

[41] Chakraborty H, Gu H (2009). A Mixed Model Approach for Intent-to-Treat Analysis in Longitudinal Clinical Trials With Missing Values.

[42] Guelinckx I, Devlieger R, Mullie P, Vansant G (2010). Effect of lifestyle intervention on dietary habits, physical activity, and gestational weight gain in obese pregnant women: a randomized controlled trial. Am J Clin Nutr.

[43] Kracht CL, Drews KL, Flanagan EW, Keadle SK, Gallagher D, Van Horn L, Haire-Joshu D, Phelan S, Pomeroy J, Redman LM (2024). Maternal 24-h movement patterns across pregnancy and postpartum: the LIFE-Moms consortium. Prev Med Rep.

[44] Sanda B, Vistad I, Sagedal LR, Haakstad LAH, Lohne-Seiler H, Torstveit MK (2017). Effect of a prenatal lifestyle intervention on physical activity level in late pregnancy and the first year postpartum. PLoS One.

[45] Sharp KJ, Sherar LB, Kettle VE, Sanders JP, Daley AJ (2022). Effectiveness of interventions to increase device-measured physical activity in pregnant women: systematic review and meta-analysis of randomised controlled trials. Int J Behav Nutr Phys Act.

[46] Sparks JR, Flanagan EW, Kebbe M, Redman LM (2023). Understanding barriers and facilitators to physical activity Engagement to Inform a precision prescription approach during pregnancy. Am J Lifestyle Med.

[47] Kracht CL, Neshteruk CD, Moding KJ, Rolke LJ, Wagner BE, Kielb E, Ferrante MJ, Robinson C, Keinsley J, Colella J, Speirs KE, Luecking CT (2024). Community-based diet and obesity-related policy, system, and environmental interventions for obesity prevention during the first 1000 days: a scoping review. Obes Rev.

[48] Melzer K, Schutz Y, Soehnchen N, Othenin-Girard V, Martinez de Tejada B, Irion O, Boulvain M, Kayser B (2010). Effects of recommended levels of physical activity on pregnancy outcomes. Am J Obstet Gynecol.

[49] Mudd LM, Owe KM, Mottola MF, Pivarnik JM (2013). Health benefits of physical activity during pregnancy: an international perspective. Med Sci Sports Exerc.

[50] Giurgiu M, Ketelhut S, Kubica C, Nissen R, Doster A, Thron M, Timm I, Giurgiu V, Nigg CR, Woll A, Ebner-Priemer UW, Bussmann JB (2023). Assessment of 24-hour physical behaviour in adults via wearables: a systematic review of validation studies under laboratory conditions. Int J Behav Nutr Phys Act.

