# Supplementary material for: Examining the Impact of a Codeveloped Multicomponent Mobile eHealth Lifestyle Intervention on Physical Activity and Its Association With Gestational Weight Gain in Underserved Women: A Statewide Randomized Controlled Trial
Source: J Med Internet Res. 2025 Nov 11;27:e73962. doi: 10.2196/73962 (PMC12648131; doi:10.2196/73962)
Supplement: Multimedia Appendix 2 [file jmir_v27i1e73962_app2.docx]

| **Multimedia Appendix 2.** Weekly content for one-on-one coaching for the intervention group and monthly one-on-one coaching for the usual care group. | | |
| --- | --- | --- |
| **Week** | **Intervention group** | **Usual care group** |
| **1** | Weight gain in pregnancy | Prenatal vitamin |
| **2** | Overcoming barriers to success | Sleep |
| **3** | Meal planning and grocery shopping | Preparing for labor and birth |
| **3** | Meal planning and grocery shopping | Preparing for labor and birth |
| **4** | Meal prep and healthy cooking | Healthy attachment |
| **5** | Portion control and eating patterns | Recommended immunization schedules |
| **6** | Behavior chains | Tooth decay in infants |
| **7** | Controlling food cues and hunger | How to take a child’s temperature |
| **8** | Building social support | Meditation for children |
| **9** | Emotional eating | Generosity in children |
| **10** | Gestational diabetes | Insect repellant |
| **11** | Protein and fat | Conflict resolution |
| **12** | Fluids and fiber | Childproofing the home |
| **13** | Carbohydrate and sugar | Cell phones |
| **14** | Prenatal vitamins | Child safety |
| **15** | Social eating | Infant learning |
| **16** | Physical activity | Car seats |
| **17** | Mindfulness and relaxation | Parenting an infant |
| **18** | Managing food cravings and snacking | Postpartum depression |
| **19** | Healthy eating on the go | Breastfeeding |
| **20** | Stress and sleep | Choosing a pediatrician |
| **21** | Postpartum depression | Back to sleep, tummy to play |
| **22** | Preparing for labor and birth | Poison safety |
| **23** | Breastfeeding | Preparing for baby |
| **24** | Optimizing health postpartum | Parenting |
