# Supplementary material for: Examining the Impact of a Codeveloped Multicomponent Mobile eHealth Lifestyle Intervention on Physical Activity and Its Association With Gestational Weight Gain in Underserved Women: A Statewide Randomized Controlled Trial
Source: J Med Internet Res. 2025 Nov 11;27:e73962. doi: 10.2196/73962 (PMC12648131; doi:10.2196/73962)
Supplement: Multimedia Appendix 3 [file jmir_v27i1e73962_app3.docx]

| **Multimedia Appendix 3.** Planned content for Facebook posts over the intervention period. | |
| --- | --- |
| **SmartTips® Topics** | Healthy Beginnings  Weight gain in pregnancy  Overcoming barriers to success  Meal planning and grocery shopping  Meal planning and grocery shopping  Meal prep and healthy cooking  Portion control and eating patterns  Behavior chains  Controlling food cues and hunger  Building social support  Emotional eating  Gestational diabetes  Protein and fat  Fluids and fiber  Carbohydrate and sugar  Prenatal vitamins  Social eating  Physical activity  Mindfulness and relaxation  Managing food cravings and snacking  Healthy eating on the go  Stress and sleep  Postpartum depression  Preparing for labor and birth  Breastfeeding  Optimizing health postpartum |
| **Diet** | Incorporate WIC approved foods in all dietary materials  8 meal plans and shopping lists  12 written recipes with photographs  12 recorded recipes  8 live cooking demonstrations |
| **Physical Activity** | Feature pregnant women in the production of all exercise materials  24 short videos/still photographs for ‘exercise of the week’  24 weeks of exercise messaging  8 live exercise classes |
