# Supplementary material for: Examining the Impact of a Codeveloped Multicomponent Mobile eHealth Lifestyle Intervention on Physical Activity and Its Association With Gestational Weight Gain in Underserved Women: A Statewide Randomized Controlled Trial
Source: J Med Internet Res. 2025 Nov 11;27:e73962. doi: 10.2196/73962 (PMC12648131; doi:10.2196/73962)
Supplement: Multimedia Appendix 4 [file jmir_v27i1e73962_app4.docx]

| **Multimedia Appendix 4.** Intervention engagement metrics. | | | | |
| --- | --- | --- | --- | --- |
|  | **Overall**  **(N=163)** | **Low Engagement**  **< 2 days per week (N=41)** | **Moderate Engagement**  **3-5 days per week (N=75)** | **High Engagement**  **>5 days per week (N=47)** |
| **Daily Weighing** |  |  |  |  |
| Average days per week | 3.7 ± 2.0 | 1.5 ± 1.2 | 3.7 ± 1.5 | 5.5 ± 1.2 |
| Percentage of max possible | 52 ± 28 | 22 ± 17 | 53 ± 22 | 78 ± 18 |
| **Recording Steps** |  |  |  |  |
| Average days per week | 3.6 ± 2.7 | 0.6 ± 0.8 | 3.5 ± 2.3 | 6.3 ± 0.9 |
| Percentage of max possible | 51 ± 38 | 8 ± 12 | 50 ± 33 | 90 ± 13 |
| **Educational Lessons** |  |  |  |  |
| Total across intervention period | 19 ± 16 | 4 ± | 18 ± 14 | 36 ± 10 |
| Percentage of max possible | 35 ± 29 | 7 ± 9 | 32 ± 25 | 64 ± 18 |
| **Health Coach Contacts** |  |  |  |  |
| Total across intervention period | 15 ± 5 | 11 ± 5 | 15 ± 5 | 18 ± 3 |
| Percentage of max possible | 84 ± 20 | 65 ± 26 | 89 ± 13 | 95 ± 7 |
| Values are means ± SD. Engagement for <2 days per week equates to less than 40% average weekly engagement; engagement for 3-5 days per week equates to 40-70% average weekly engagement; Engagement for 5 days per week equates to at least 70% average weekly engagement. Percentage of max possible was calculated as the proportion of days participants self-weighed or synced their fitness tracker to record daily steps relative to the entire intervention period (24 weeks or until delivery), the proportion of lessons watched out of all that were available, and the coach contacts out of total attempts made | | | | |
