# Supplementary material for: Examining the Impact of a Codeveloped Multicomponent Mobile eHealth Lifestyle Intervention on Physical Activity and Its Association With Gestational Weight Gain in Underserved Women: A Statewide Randomized Controlled Trial
Source: J Med Internet Res. 2025 Nov 11;27:e73962. doi: 10.2196/73962 (PMC12648131; doi:10.2196/73962)
Supplement: Multimedia Appendix 5 [file jmir_v27i1e73962_app5.docx]

| Multimedia Appendix 5. Device-measured physical activity levels during early and late pregnancy per randomization assignment and BMI category. | | | | | | | |
| --- | --- | --- | --- | --- | --- | --- | --- |
|  | **Intervention** | | | **Usual care** | | |  |
|  | **Early Pregnancy** | **Late Pregnancy** | **Within Group**  **Change** | **Early Pregnancy** | **Late Pregnancy** | **Within Group Change** | **Between Group Change** |
| Inactive time, min/day |  |  |  |  |  |  |  |
| Normal Weight | 712 [683; 741] | 751 [716; 785] | 39 [3; 75],* ^a^  P=0.04 | 706 [677; 735] | 765 [730; 800] | 59 [23; 94]* | P=0.44 |
| Overweight | 688 [659; 717] | 738 [698; 777] | 50 [10; 89],*^a,b^ | 677 [647; 707] | 732 [696; 769] | 56 [18; 94]* | P=0.82 |
| Obesity | 696 [670; 722] | 774 [743; 806] | 89 [57; 120],*^b^ | 678 [653; 704] | 716 [684; 748] | 40 [6; 75]* | **P=0.04** |
| Light activity, min/day |  |  |  |  |  |  |  |
| Normal Weight | 174 [159; 188] | 181 [165; 198] | 8 [-10; 26] | 179 [165; 193] | 167 [150; 184] | -12 [-30; 6] | P=0.12 |
| Overweight | 187 [173; 201] | 196 [177; 216] | 9 [-11; 29] | 192 [177; 206] | 189 [171; 206] | -3 [-22; 16] | P=0.37 |
| Obesity | 185 [172; 197] | 179 [163; 194] | -6 [-22; 10] | 196 [184; 208] | 192 [175; 209] | -4 [-22; 13] | P=0.89 |
| Moderate activity, min/day |  |  |  |  |  |  |  |
| Normal Weight | 66 [55; 76] | 60 [48; 72] | -6 [-18; 6] | 77 [67; 87] | 63 [51; 75] | -14 [-26; -2]* | P=0.36 |
| Overweight | 82 [71; 93] | 66 [52; 80] | -16 [-30; -3]* | 83 [72; 93] | 78 [65; 90] | -5 [-18; 8] | P=0.24 |
| Obesity | 75 [66; 85] | 60 [48; 71] | -16 [-27; -5]* | 84 [75; 93] | 74 [62; 86] | -10 [-22; 1] | P=0.49 |
| Vigorous activity, min |  |  |  |  |  |  |  |
| Normal Weight | 1 [0; 3] | 1 [0; 3] | 0 [-2; 2] | 2 [1; 3] | 2 [0; 4] | 0 [-2; 2] | P=0.91 |
| Overweight | 2 [1; 3] | 1 [-1; 3] | -1 [-3; 2] | 2 [1; 4] | 2 [0; 4] | 0 [-3; 2] | P=0.76 |
| Obesity | 3 [2; 4] | 1 [-1; 2] | -2 [-4; 0]* | 2 [1; 3] | 1 [0; 3] | 0 [-3; 2] | P=0.21 |
| MVPA, min |  |  |  |  |  |  |  |
| Normal Weight | 67 [56; 77] | 61 [48; 73] | -6 [-18; 7] | 79 [68; 89] | 65 [52; 78] | -14 [-26; -2]* | P=0.36 |
| Overweight | 84 [73; 95] | 67 [53; 82] | -17 [-31; -3]* | 85 [74; 96] | 80 [66; 93] | -5 [-19; 8] | P=0.23 |
| Obesity | 78 [69; 88] | 60 [48; 71] | -19 [-30; -8]* | 86 [76; 95] | 75 [63; 88] | -11 [-23; 2] | P=0.34 |
| MVPA, bouts, 1-5 min |  |  |  |  |  |  |  |
| Normal Weight | 12 [9; 16] | 11 [7; 16] | -1 [-5; 4] | 16 [13; 20] | 12 [8; 17] | -4 [-8; 0] | P=0.30 |
| Overweight | 18 [14; 21] | 11 [6; 16] | -6 [-11; -2]* | 18 [14; 22] | 15 [11; 20] | -3 [-7; 2] | P=0.28 |
| Obesity | 15 [12; 18] | 9 [5; 13] | -6 [-10; -2]* | 19 [15; 22] | 13 [9; 17] | -6 [-10; -1]* | P=0.91 |
| Data presented as estimated marginal means alongside corresponding 95% confidence intervals with within- between-group effect estimates and p-values derived from a linear mixed model including fixed effects for time and group as well as the interaction thereof, and a random effect for participant. Model adjusted for body mass index category at enrolment. *, within-group change p<0.05. ^a, b, c^ superscripts denote a statistically significant difference between BMI categories. MVPA = moderate-to-vigorous physical activity | | | | | | | |
