# Supplementary figures and images for: Examining the Impact of a Codeveloped Multicomponent Mobile eHealth Lifestyle Intervention on Physical Activity and Its Association With Gestational Weight Gain in Underserved Women: A Statewide Randomized Controlled Trial
Source: J Med Internet Res. 2025 Nov 11;27:e73962. doi: 10.2196/73962 (PMC12648131; doi:10.2196/73962)

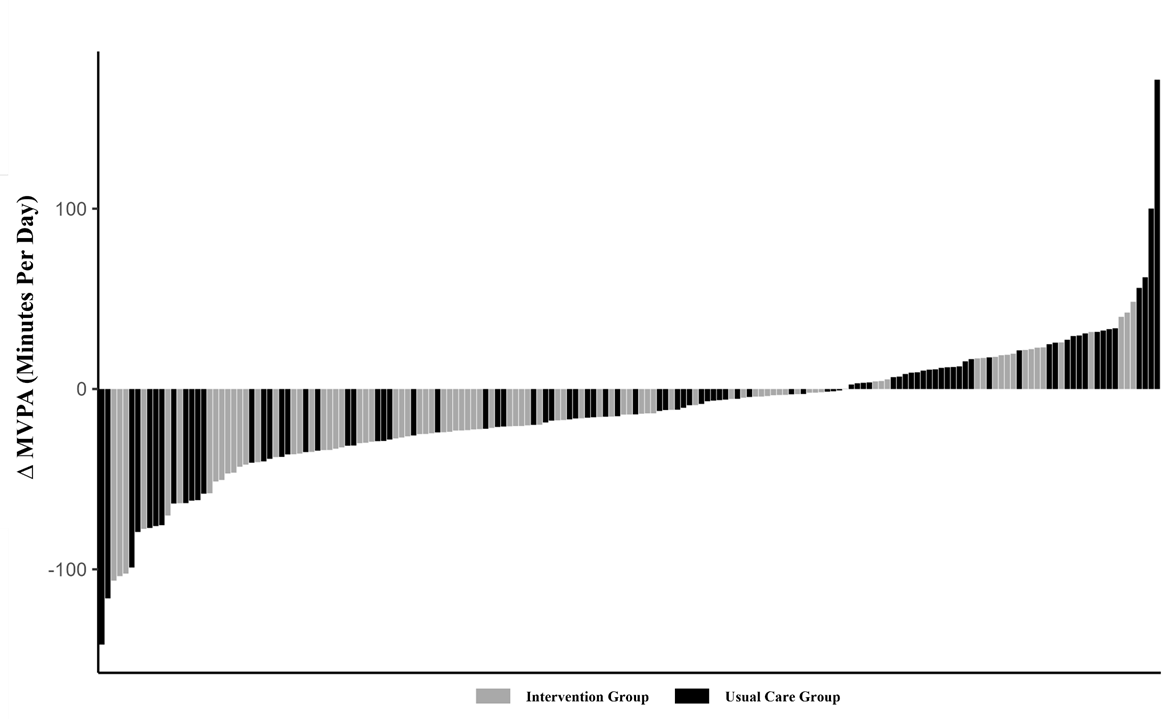

Supplement: Multimedia Appendix 6 [file jmir_v27i1e73962_app6.png]
